# Novel Approach for the Preparation of a Highly Hydrophobic Coating Material Exhibiting Self-Healing Properties

**DOI:** 10.3390/molecules29163766

**Published:** 2024-08-09

**Authors:** Uwe Holzdörfer, Wael Ali, Eckhard Schollmeyer, Jochen S. Gutmann, Thomas Mayer-Gall, Torsten Textor

**Affiliations:** 1 Deutsches Textilforschungszentrum Nord-West gGmbH, Adlerstr. 1, 47798 Krefeld, Germany; 2 Physikalische Chemie, Center for Nanointegration Duisburg-Essen, Universität Duisburg-Essen, Universitätsstraße 2, 45117 Essen, Germany; 3 TEXOVERSUM School of Textiles, Reutlingen University, Alteburgstr. 150, 72762 Reutlingen, Germany

**Keywords:** self-healing, hydrophobicity, sol-gel, silica nanoparticle

## Abstract

A concept to prepare a highly hydrophobic composite with self-healing properties has been designed and verified. The new material is based on a composite of a crystalline hydrophobic fluoro wax, synthesized from montan waxes and perfluoroethylene alcohols, combined with spherical silica nanoparticles equipped with a hydrophobic shell. Highly repellent layers were prepared using this combination of a hydrophobic crystalline wax and silica nanoparticles. The novel aspect of our concept was to prepare a ladder-like structure of the hydrophobic shell allowing the inclusion of a certain share of wax molecules. Wax molecules trapped in the hydrophobic structure during mixing are hindered from crystallizing; therefore, these molecules maintain a higher mobility compared to crystallized molecules. When a thin layer of the composite material is mechanically damaged, the mobile wax molecules can migrate and heal the defects to a certain extent. The general preparation of the composite is described and XRD analysis demonstrated that a certain share of wax molecules in the composite are hindered to crystallize. Furthermore, we show that the resulting material can recovery its repellent properties after surface damage.

## 1. Introduction

In the late 1940s, Wenzel [1,2,3] as well as Cassie and Baxter [4,5] explained the influence of the topography of a surface with a given chemical composition on repellence. The Cassie–Baxter model predicts an increase in apparent contact angle with the increasing roughness of an inherently hydrophobic surface. Optimal repellence can be achieved with structures featuring both micro- and nanometer scale. Since that time, scientists have been striving to develop hydrophobic surfaces with minimal surface energy combined with specific surface structures to create super-repellent surfaces [6,7,8,9,10,11,12]. The primary challenge is to prepare a durable hydrophobic surface structure in the micrometer range. Maximum repellence will be achieved when an additional structure in the nanometer range is also incorporated.

Various approaches have been investigated leading to more or less successful results. A significant issue with structured hydrophobic surfaces is their comparatively low mechanical resistance. During use, the super-repellent surfaces can become abraded, resulting in flat hydrophobic surfaces at best, and hydrophilic surfaces at worst if the abrasion not only flattens the structure but also removes the hydrophobic coating completely [13]. Different plant waxes are suitable to recover the wear [14]. As a biomimetic approach, nanotechnology has emerged as a promising approach to develop micro- and possibly nanostructured hydrophobic surfaces with improved wear resistance [15,16,17,18]. The nanoparticles employed are expected to improve the abrasion resistance and can also serve as shape-forming templates to achieve a desired topography.

As most approaches employing nanoparticles for the preparation of super-repellent surfaces are focused on an improvement in the wear-resistance, the authors’ superior aim was to develop novel composites capable of recovering the super-repellent properties if damaged by abrasion. The basic approach was to prepare a composite based on inorganic nanoparticles with surfaces modified by long-chained highly fluorinated alkoxysilanes, combined with a new extremely hydrophobic highly crystalline wax system [15,16,17,18].

The inclusion of a certain share of wax into hydrophobic structures on the particle surface will prevents the crystallization of these wax molecules and guarantee a degree of mobility (compared to the crystalline share of wax) of the hydrophobic material in case of a physical damage to the super-repellent surface. This mobility allows for a certain recovery of the damaged layer. Thus, the combination of hydrophobic nanoparticles and hydrophobic wax guarantees highly or even super-repellent surfaces. Self-healing can be achieved if the mobility of the wax molecules, hindered from crystallizing by inclusion into the hydrophobic structure, is sufficient to close—“repair”—damaged areas. 

This approach is also applied by different other authors. Different waxes [19,20,21], bio-based or fluor-containing systems, have been used to obtain hydrophobic and self-repairing coatings. A fluorine-free approach demonstrates that self-healing and hydro- or superhydrophobicity function depends on the particle size and the particle-to-wax ratio [22]. Other approaches use self-lubricating slippery surfaces, which store a hydrophobic solvent in a nanoporous surface. The stored solvent prevents the surface from damaging [23,24,25,26,27]. By using dynamic covalent bond chemistry or vitrimers also, self-healing hydrophobic surfaces are possible [28,29,30]. All self-healing approaches have in common that these processes take a certain time or need activation energy in the form of heat or light.

Self-healing hydrophobic surfaces are interesting not only for the mentioned example of a high-class mountaineering cord. In applications where material can suffer abrasive damage like outdoor clothes, roofing membranes, personal protective equipment like work wear or tents, these properties are highly desired.

The hydrophobic highly crystalline wax system used in this study was synthesized based on the so-called montan waxes. These montan waxes [31,32,33,34,35] are byproducts of brown coal mining that are separated from coal by extraction. The raw product is basically a mixture of long-chained fatty acid esters and alcohols (C_22_–C_34_) and has been characterized in a very detailed manner within the framework of the overarching aim of the underlying research project. Following our approach, the montan waxes have to be purified, the esters have to be saponified and the long-chained alcohols have to be oxidized to yield a mixture of the corresponding long-chained, unbranched fatty acids with a high purity. Alternatively, a commercially available material (Luwax^®^ S, BASF) has been used. An extremely hydrophobic crystalline wax was synthesized by esterification of the acids with perfluoroalkylethyl alcohols (C_8_–C_12_). While a more detailed description of the wax and its preparation is not necessary here, it is important to note that the novel approach for preparing self-healing material relies on using a hydrophobic wax based on linear molecules with a strong tendency to crystallize.

## 2. Results and Discussion

### 2.1. Preparation of Hydrophobic Nano Particles

**Figure 1:**
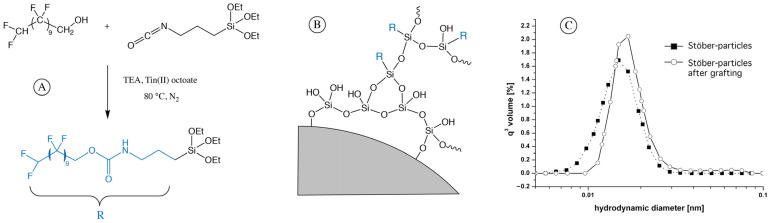
(**A**) Reaction scheme for the synthesis of the fluorinated alkoxy silane. (**B**) Modification of the silica particle surface. (**C**) DLS measurement of Stöber particles before and after grafting of fluorinated silane.

One of the ideas of our approach was to employ nanoparticles modified with hydrophobic surface groups that allow and facilitate the inclusion of wax molecules, thus preventing their crystallization. For the first investigations, Stöber particles [36] were employed and modified with a long-chained, highly fluorinated alkoxysilane. Commercially available silanes typically have relatively short fluorocarbon chains (maximum C_6_). However, lower surface energies as well as a more effective inclusion of wax molecules are expected using hydrophobic silanes with longer chains. To this end, a silane with a C10-fluorocarbon chain was synthesized by reflux heating 3-isocyanatopropyltriethoxysilane and *α*,*α*,*ω*-trihydroperfluoroundecanol in the presence of triethanolamine and tin(II) octoate at a temperature of 80 °C under a nitrogen atmosphere (Figure 1A).

Silica particles in methyl-2-pentanone and the synthesized alkoxysilane were mixed prior to the addition of hydrochloric acid, which served as a catalyst to initiate the grafting reactions (Figure 1B). The silanol groups on the surface of the silica particles were quantified following the method described by Gorlov [37], which allowed to set a one-to-one ratio between silanol groups and the synthesized alkoxysilane. The grafting process was carried out by refluxing the mixture for 48 h. Subsequentially, small amounts of precipitates were filtered off before the addition of hexamethyldisilazane, which was used to end-cap any remaining silanol groups, thus enhancing the ageing stability of the sol. Ideally, the grafting reaction would result in a monomeric coverage. However, the formation of “ladder structures” is anticipated to be more realistic, although it has not been proven here. For silica particles of an initial size of 15.4 nm, dynamic light scattering (DLS) measurements indicated an increase in hydrodynamic diameter of the particle by approximately 1.6 nm (Figure 1C).

### 2.2. Preparation of the Highly Repellent Wax

**Figure 2:**
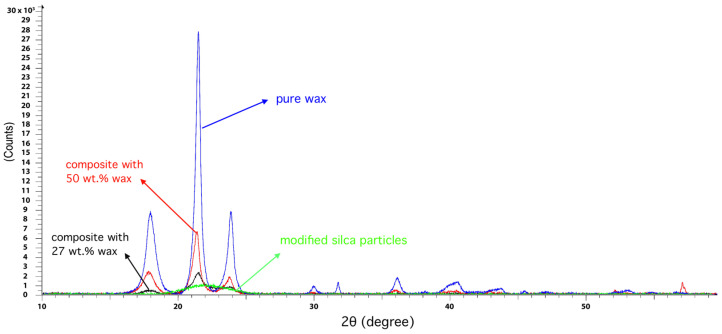
XRD spectra of the pure wax, the modified silica nanoparticles and two composites containing different amounts of wax. (broad, flat spectrum: silica nanoparticles only, the other spectra represent measurements for composites with 27, 50 and 100 wt.% wax—the higher the intensity the more the share of wax).

The wax used to prepare highly repellent surfaces was synthesized from montan waxes, which are naturally occurring mixtures of C_22_–C_34_ fatty acids and alcohols. The linear structure of these acids and alcohols allows the formation of crystalline waxes. Commercially available purified montan waxes, which contain only the fatty acids, provide a certain repellence but only for more polar liquids. To enhance repellent properties, fluorocarbon groups were introduced via esterification. Several esterification processes were tested, but satisfactory yields were only achieved when catalyzing the reaction with tetrabutyl titanate [35], resulting in ~97% yield. Following the distillation of the byproduct water and cooling to ambient temperature, a pale waxy material was obtained. As supposed, XRD measurements confirmed a strong tendency of the resulting ester to crystallize, as evidenced by the prominent peaks observed in Figure 2.

### 2.3. Preparation and Application of the Composite

**Figure 3:**
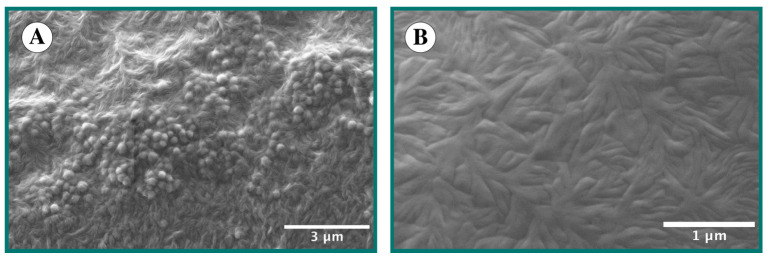
SEM micrographs of a composite prepared from the fluorinated wax and modified silica particles of approx. 15 nm (**B**) and corresponding particles of 286 nm (**A**).

The composite material was prepared in a simple manner by mixing the fluorinated wax with the sol containing the modified silica nanoparticles using methyl-2-pentanone as a solvent. The desired ratios of wax to sol were achieved by stirring the mixture while heating it to 60 °C, yielding a clear solution. This solution was then applied to glass slides (as model substrate) as well as textile substrates through casting and padding, respectively. The samples were subsequently dried to remove the solvent. The SEM images of two composites prepared with silica particles of different sizes are shown in Figure 3. The micrographs reveal that the composite with very small silica particles (~15 nm) displays a topography indicative of a strong tendency for the material to crystallize. In contrast, composites prepared with larger particles (~286 nm) exhibit a microstructured surface.

### 2.4. Investigation of the Expected Inclusion of Wax Molecules

XRD measurements were carried out with selected samples to investigate the potential intercalation of wax molecules between the hydrophobic surface groups of the silica particles. XRD spectra were recorded of the silica particles (after surface modification with fluorocarbon groups), the pure wax (after esterification reaction with fluorocarbon groups) and two composites with different shares of nanoparticles. The corresponding XRD spectra are shown in Figure 2.

**Figure 4:**
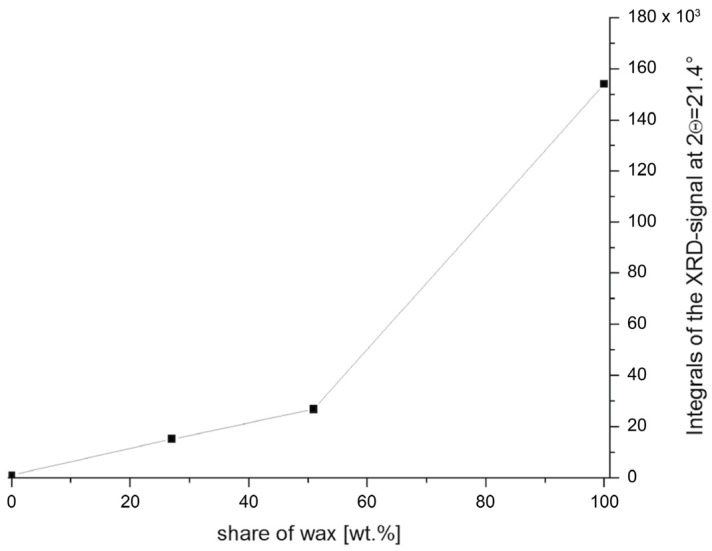
Graphical analysis of the integral of diffraction peaks at 2θ = 21.4° as a function of the wax proportion in the investigated materials (solid line is guide to eyes).

As expected, the amorphous silica particles show no diffraction peaks, while the fluorinated wax shows three strong signals. Adding certain amounts of nanoparticles to the wax resulted in a decrease in signal intensity, due to the reduction in the proportion of crystalline material forming the composite. A more detailed analysis of the integrated signal intensities is shown in Figure 4. The data reveal that the integrals of the signal for the nanoparticle–wax composites, for a given amount of wax, are smaller than those of the pure wax. This suggests that the wax crystallizes to a lower degree when the composite is prepared, which aligns well with our expectations.

The aim of this study was to develop a hydrophobic material. Therefore, the surface energy and contact angle of the resulting materials are of fundamental importance concerning the desired properties. Surface energy was calculated following the approach of Owens and Wendt [38]. Contact angles were measured for water (143.0° ± 0.1) and for diiodomethane (132.4.0° ± 0.2) on coatings applied to glass slides. The SEM of the coated surface (see Figure 3A) reveal that the surface is not flat, which explains the discrepancies in the contact angle measurements, as the Owens and Wendt method requires measurements on flat surfaces, which is not possible for the composite investigated here. Nevertheless, the results demonstrate that the composite exhibits low surface energy and an increased repellence, attributable to both surface energy and the surface structure. The roughness of the coatings was investigated by atomic force microscopy (AFM) measurements, with the roughness (R_a_) calculated to be 200 nm.

**Figure 5:**
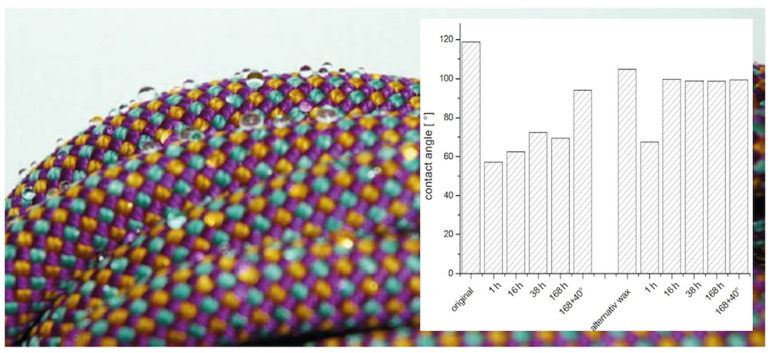
Water droplets on a rope and their contact angle measured after plasma etching and subsequent coating recovery over different time durations. A comparison was made between the original system and an alternative wax.

An important aspect of this study was the self-healing ability of the highly hydrophobic layer. To evaluate this property, several technical products were coated, and the surfaces were damaged via plasma etching. The commercial product finished with two composites was a high-class mountaineering cord. These cords require high repellence, as a water-soaked rope significantly increases the risk of tearing when the climber falls dramatically. The repellent coating must withstand damage from friction during use, as defects ingress via capillary forces. Ropes coated with the hydrophobic composite were subjected to plasma etching, and the contact angle after recovery was monitored (see Table 1 and Figure 5). The contact angle of about 119° before etching, was reduced to 57° due to the plasma process. The recovery of the contact angle within 168 h was less than expected, reaching only a value of 72.5°. However, additional tempering at 40 °C resulted in a notable recovery and a contact angle of at least 94.2° was achieved. This indicates that the mobility of the wax molecules is insufficient for recovery within a relevant timescale under ambient conditions.

To validate the general concept, an alternative composite was prepared by replacing the wax with a less crystalline one, prepared from 2-butyloctanoic acid and the perfluoroethylene alcohol. The branched alkyl rest is expected to reduce the crystallinity of the resulting wax. The contact angle achieved by the alternative composite was approximately 104.8°, which is lower than that of the previously tested composite tested (see Table 1). Plasma etching again resulted in a significantly decreased contact angle for one hour in recovery. However, the alternative composite showed a relatively fast and nearly complete recovery, achieving a stable contact angle (~100°) from 16 h onward. While plasma etching provides valuable information on the recovery ability of the treated surfaces, it is important to note that etching not only causes physical damaging but also leads to oxidation, increasing the hydrophilicity of the wax material at the surface. Finished ropes were also subjected to realistic operational tests, e.g., a treatment with a so-called rope-tester, which simulates wear damage. The product showed comparable performance in this specific test. The self-healing effect works by the inclusion of a certain fraction of wax into hydrophobic structures on nanoparticle surfaces. This will prevent the crystallization of these wax molecules, whereby the degree of mobility of the hydrophobic material is increased. In case of a physical damage event, this mobility allows a recovery of the damaged layer, which is observed by regaining a higher contact angle after a certain time after the damaging of the surface.

From the authors’ point of view, the results support the basic concept for a self-healing repellent material that was followed here. Future investigation will be needed to describe the alternative composite in more detail as presented for the one based on montan wax. Celik et al. demonstrated a similar but fluorine-free and bio-based approach based on carnauba wax. In this case, the right particle size and nanoparticle-to-wax ratio had to be selected to obtain a self-healing and hydrophobic surface [22].

Besides being a scientifically interesting system, it can be stated that the ropes finished with the described composites outperform commercially available products.

## 3. Materials and Methods

### 3.1. Materials

3-Isocyanatopropyltriethoxysilane (GE-Crompton, >95%) was purified by vacuum distillation to remove impurities. *α*,*α*,*ω*-trihydroperfluoroundecanol supplied by Halogen Russia (Perm) (92.0%) was also purified via vacuum distillation before use. The following substances were used without further purification: ammonia 25% p.a. (Fluka, Buchs, Switzerland), 2-Butyl-1-octanol (Isofol 12, >97% Sasol, Hamburg, Germany), ethanol (>99.9%; H_2_O: <0.05% Alcosuisse, Rüti bei Büren, Switzerland), hexamethyldisilazane (HMDS, >98%, Fluka), hexane (>99.0%, Fluka), tetraethoxysilane (TEOS, >99.9%), tin(II), octoate (Metatin S 28, Rohm & Haas, Acima, Buchs, Switzerland), MIBK (4-Methyl-2-pentanone, >98%), Fluka and Luwax S (BASF, San Bruno, CA, USA)).

### 3.2. Analytic

Infrared spectra were obtained by preparing KBr samples and measured using a Perkin Elmer (Waltham, MA, USA) 1650 spectrometer. NMR-spectra were recorded with a Bruker DPX 400. The ^1^H-NMR spectra were recorded at 400 MHz using CDCl_3_ as a solvent, while the ^13^C-NMR spectra were recorded at 100 MHz using the same solvent. XRD investigations were performed using a Siemens (Bruker (Billerica, MA, USA) AXS) D 5005 instrument with an OED Detector. The X-ray source was copper, and the frequency used was 0.154056 nm (Cu-K_α_). Contact angles were measured using a Krüss DSA 10 with Drop Shape Analysis Software DSA 1 v 1.9. The testing liquids were distilled water and diiodomethane (Fluka (Geel, Belgium) AN 66880; purum > 98.0%), the volume of the drops was 10 μL and the deposition of a drop was carried out with 50 μL/min. Particle size was evaluated via dynamic light scattering (DLS) using a Zetasizer Nano S (Malvern Instruments Limited, Malvern, UK).

SEM micrographs were prepared with a Zeiss Supra 40 VP. Fluorine content of the products was determined by combustion of the dried samples according to the Schöniger method [39], followed by ion-sensitive fluoride determination.

To determine the surface roughness, atomic force microscopy (AFM) measurements were performed in contact imaging mode using an Agilent Technologies (Santa Clara, CA, USA) model 5500 beam-deflection atomic force microscope. Nanosensors with standard contact-mode cantilevers and a resonance frequency of 13 kHz, force constant of 0.2 N/m, length of 450 μm, width of 50 μm and thickness of 2 μm were used. The sampling resolution of all the images was set at 1024 data points with a scanning speed of 0.5 lines/s. All the captured images were processed using the open-source Gwyddion 2.49 software, and the arithmetic average roughness (Ra) was obtained by averaging the row/column roughness statistics of the whole image.

### 3.3. Plasma Etching

Plasma etching was carried out in a plasma chamber purged with argon/oxygen (1:1; *v*/*v*, flow rate: 25 ccm/min, pressure 10^−4^ bar). The RF-plasma operated at an excitation frequency of 13.56 MHz with a power of 40 W. Samples were treated for 10 min.

### 3.4. Preparation of Stöber Particles

Stöber particles were prepared by dissolving TEOS in ethanol, followed by the addition of specific amounts of ammonia and distilled water at room temperature. After initiating the hydrolysis, the sols were stirred for at least 16 h in a closed vessel. After preparation, the sols were concentrated by partially evaporating the solvent. For the preparation of 16 nm particles, 35.5 g (0.170 mol) TEOS was dissolved in 770 g EtOH, and 35.0 g of 25% aqueous NH_3_ was slowly added.

### 3.5. Preparation of the Hydrophobic Silane

**Figure 6:**
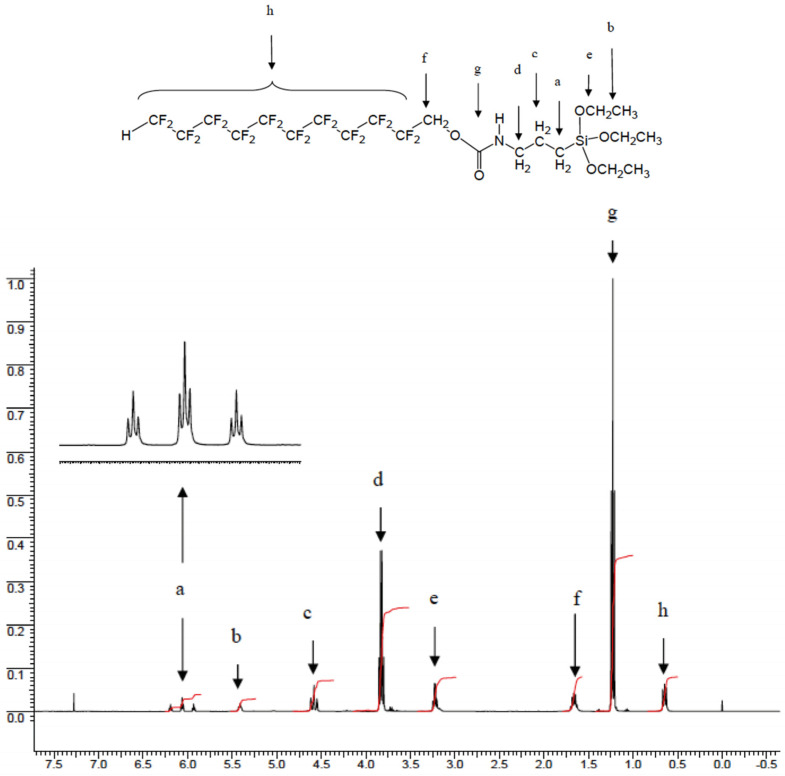

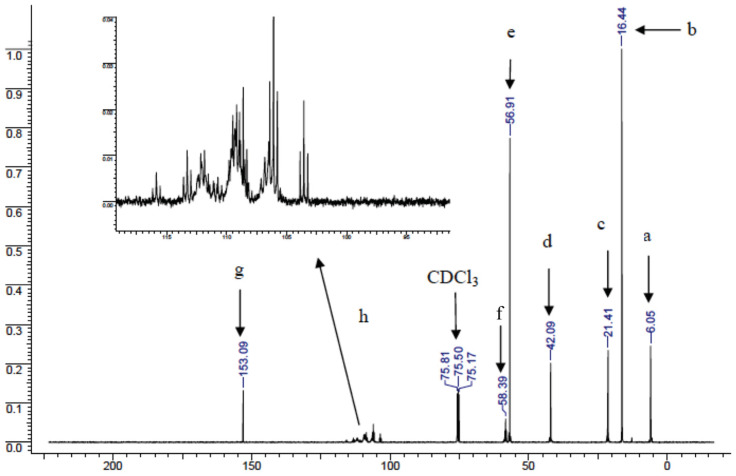
^1^H- and ^13^C-NMR spectra of the fluorinated silane.

For preparation of the fluorinated silane (see Figure 1A), *α*,*α*,*ω*-trihydroperfluoroundecanol and 3-Isocyanatopropyltriethoxysilane were mixed in an equimolar ratio. Additionally, triethanolamine and tin octoate were added. The reagents were melted and kept at 80 °C in a nitrogen atmosphere for at least one hour. The product of the reaction was a colorless greasy wax, which was used without further purification. The fluorine content was measured as 44.1%, which is in good agreement with the theoretical value of 44.9%. An infrared spectrum of the product did not show any signals indicating residual isocyanato functions. The further NMR (Figure 6) and IR data are summarized below.

^1^H-NMR: d_H_ (400 MHz; CDCl_3_; Me_4_Si): 6.06 (3t, 1H, H-CF_2_-, ^2^J_H-CF2-CF2-_ = 51.8; ^3^J _HCF2-CF2-_ = 5.04); 5.41 (b, 1H, -NH); 4.58 (m, 2H, -CF_2_-CH_2_-O-); 3.83 (m, 2H, -O-CH_2_-CH_3_); 3.22 (m, 2H, -NH-CH_2_-); 1.67 (m, 2H, -CH_2_-CH_2_-CH_2_-); 1.23 (t, 2H, -CH_3_); 0.64 (m, 2H, -CH_2_-Si).
^13^C-NMR: d_C_ (100 MHz, de-coupled, CDCl_3_, Me_4_Si): 6.05 (-CH_2_-Si-); 16.44 (-CH_3_); 21.41 (-NH-CH_2_-CH2-); 42.09 ((-NH-CH_2_-CH_2_-); 56.91 (Si-O-CH_2_); 58.39 (-CF_2_-CH_2_-O-) ^2^J_FC-CH2-_ = 104); 103–116 not resolved (HCF_2_-C_10_F_20_-); 153.09 (C=O).
IR (KBr molding film): ν_max_/cm^−1^ 3348w (NH); 2978w (CH_3_), 2932w (-CH_2_-); 2889w (-CH_2_-); 1732w (-C=O); 1533w (-C-N); 1445w (-CH_2_-); 1380w (-CH_3_); 1206s (-C=O); 1145s (-CF_2_-); 1103s (-CF_2_-); 770w (-CF).

### 3.6. Surface Grafting of Stöber Particles with the Hydrophobic Silane

**Scheme 1:**
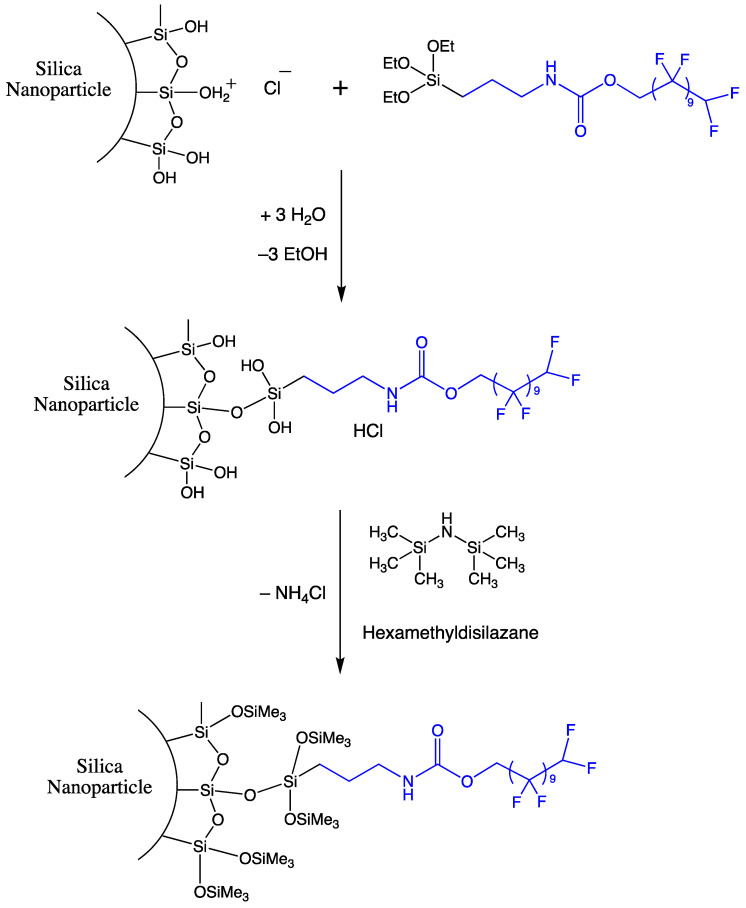
Surface modification of silica nanoparticles with the synthesized fluorinated silane.

The surface-grafting of the silica particles was carried out by adding the previously synthesized alkoxysilane to the different silica sols (Scheme 1). The ratio of available silanol groups to added silane was approximately one-to-one. The silanol groups were determined via a method described by Gorlov et al. [37], which involves the chlorination of the silanol groups with thionyl chloride and a subsequent determination of the chloride content. The analysis of the fluorine content of the modified nanoparticles, after the removal of all solvents, showed a total fluorine content of 11.53% for the modified particles with a diameter of 15 nm. This value represents about 94.5% of the fluorine content calculated from the number of silanol groups theoretically available for condensation with the silane. Since the grafting with the comparably voluminous silane molecule will not remove all residual hydroxyl groups on the particle surface, and simultaneously not all silanol groups of the employed alkoxysilanes will crosslink due to steric reasons, an end-capping of the silanol functions can be carried out, e.g., to prevent an unwanted aging of the sols. The end-capping was performed using hexamethylsilazane.

### 3.7. Preparation of a Wax Based on Luwax S and the Perfluoroethylene Alcohol (Fluowet EA 812 AC)

To begin, 326.48 g (800 mmol) Luwax S (refined mixture of carbon acids from montan wax), 429.24 g (840 mmol) Fluowet EA 812 AC, 1.01 g sodium hypophosphite monohydrate and 3.78 g tetrabutyl orthotitanate were heated at 180 °C for 4 h in a three-necked flask equipped with a Dean–Stark apparatus. After 4 h, an additional 2 g tetrabutyl orthotitanate was added. The mixture was allowed to cool to 160 °C before adding 2 g NaCl solution. The dark mixture lightened immediately, yielding 703 g (97% of the theoretical yield) of a light-yellow wax with an acid number of 9.4 mg KOH/g.

### 3.8. Preparation of an Alternative Wax Based on 2-Butyloctanoic Acid and the Perfluoroethylene Alcohol

**Scheme 2:**
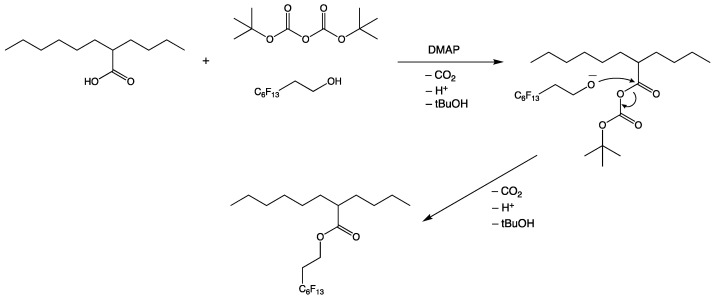
Synthesis of the perfluoroethyl wax.

A mixture of 3.64 g (10 mmol) 3,3,4,4,5,5,6,6,7,7,8,8,8-Tridecafluorooctanol and 2.00 g (10 mmol) of 2-Butyloctanacid were dissolved in 20 mL n-hexane. Then, 80 mg 4-dimethylaminpyridine and 2.84 g (13 mmol) di-tert-butyl dicarbonate were added to the mixture. The reaction mixture was stirred at 50 °C for three hours. Following this, 30 mL n-hexane was added before the mixture was transferred into a separating funnel and washed twice with 15 mL aqueous HCl (7 wt.%), twice with 15 mL aqueous NaCO_3_ solution (10 wt.%) and twice with water. The organic phase was dried with 3 g of magnesium sulfate and then reduced using a rotary evaporator, before it was dried at ambient temperature and a pressure of 10^−5^ bar until no change in weight was observed. This process yielded 4.98 g (91% of the theoretical yield) of a colorless oil. Scheme 2 show the reaction.

^1^H-NMR (400 MHz; CDCl_3_; ppm): 4.37 (m, 2H, C_6_F_13_-CH_2_-CH_2_-; ^3^J = 8); 2.51 (m, 2H, C_6_F_13_-CH_2_-CH_2_-) 2.35 (2t, 1H, -CH-COOH); 1.49 (m, 2H, C_3_H_7_-CH_2_-); 1.45 (m, 2H, C_5_H_11_-CH_2_-); 1.29 (m, 12H, -CH_2_-); 0.88 (m, 6H, -CH_3_).
^13^C-NMR (100 MHz, decoupled, CDCl_3_; ppm): 12.49 (CH_3_-C_3_H_6_-); 12.65 (CH_3_-C_5_H_10_-); 21.21 (CH_3_-CH_2_-C_4_H_8_-); 21.24 (CH_3_-CH_2_-C_2_H_4_-); 26.03 (C_4_H_9_-CH_2_-CH_2_-); 27.85 (C_5_H_11_-CH_2_-CH-); 29.30 (C_2_H_5_-CH_2_-C_2_H_4_-); 28.25 (C_3_H_7_-CH_2_-CH-); 30.55 (C_3_H_7_-CH_2_-C_2_H_4_-); 31.03 (C_2_H_5_-CH_2_-C_3_H_6_-); 44.28 (CH Methine); 57.34 (Rf-CH_2_-CH_2_-); 106–118 (C_6_F_13_-); 174.87 (-C=O).
IR (KBr liquid film; cm^−1^): 2959 (s) ν_as_ -CH_3_; 2932 (s) ν_as_ -CH_2_-; 2861 (s) ν_s_ -CH_2_-; 1740 (s) ν -C=O carboxylic acid esters; 1464 (m) ν_as_ -CH_3_; 1458; ν_s_ -CH_2_-; 1240 (s) ν -C-O- ester; ν -CF_3_; 1206 (s) ν -CF_3_; 1164 (w) ν -CF_2_-; 1145 (m) ν -CF_2_-; 733 (w) ν -CH_2_- rocking; 709 + 700 (w) ν -CF.

### 3.9. Preparation and Investigation of the Composites

The composites were prepared by mixing the modified nanoparticles (directly from the sol) and the wax in the desired ratio. The mixture was stirred and heated to 80 °C until a clear solution was obtained. For application, the composite can be further diluted with the solvent (additional dispersant). For investigation of the resulting products, the mixture was simply dried to remove the solvent.

### 3.10. XRD Measurements

XRD measurements of the pure wax system are depicted in Figure 2 (blue), proving that the wax is a highly ordered system. In the investigated area, three signals can be observed between 2θ = 15 and 25. Notably, Lorenz investigated comparable systems [40] and described three identical peaks in this region (he investigated perfluorinated dendrimers based on C8-alkyl groups). Lorenz reported that these peaks indicate a layered structure of the perfluoro groups. The comparatively broad peak at 2θ = 17.8° can be explained by the interference of the perfluoroalkyl chains. Lorenz calculated a distance of 4.9 Å between the chains of the dendrimer he synthesized and the signal position identical to that in this work. However, Lorenz reported a half-width of 10 on the 2θ scale, whereas in this work, it was only 1. This indicates the formation of larger crystallites in this work (crystallite size is calculated to be 83.5 Å). The peaks at 21.4° and 24.0° are assigned to alkyl groups.

## Figures and Tables

**Table 1:** The contact angle within 1 to 168 h after plasma etching of the repellent composite surfaces. The evaluation was performed for the combination of the modified particles with two different waxes. The change in contact angle before and after various durations following surface etching is given.

	As Prepared	1 h	16 h	38 h	168 h	168 h + Storage at 40 °C
original (high crystallin)	119	57	62	73	70	94
alternative wax (low crystallin)	105	68	100	100	99	100

## Data Availability

The data are not publicly available.
